# Supplementary material for: Hypoxia Downregulates LPP3 and Promotes the Spatial Segregation of ATX and LPP1 During Cancer Cell Invasion
Source: Cancers (Basel). 2019 Sep 19;11(9):1403. doi: 10.3390/cancers11091403 (PMC6769543; doi:10.3390/cancers11091403)
Supplement: Supplementary file 1 [file cancers-11-01403-s001.pdf]

Supplementary Materials: Hypoxia Downregulates LPP3 and Promotes the Spatial Segregation of ATX and LPP1 During Cancer Cell Invasion

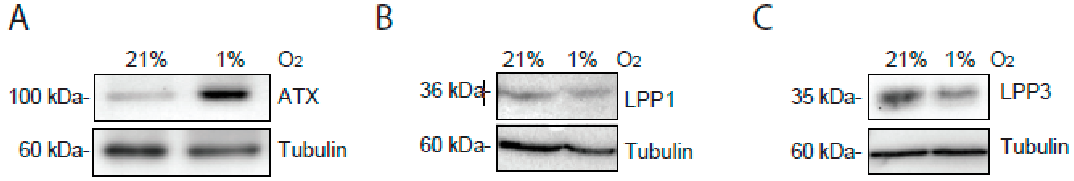

Supplementary Figure 1. Influence of hypoxia on ATX, LPP1, and LPP3 protein expression. HT-1080 cells were incubated overnight under normoxic (21% O_2) or hypoxic conditions (1% O_2) for 16 h and protein levels of (A) ATX, (B) LPP1 or (C) LPP3 were analyzed by western blotting. Tubulin was used as a loading control.

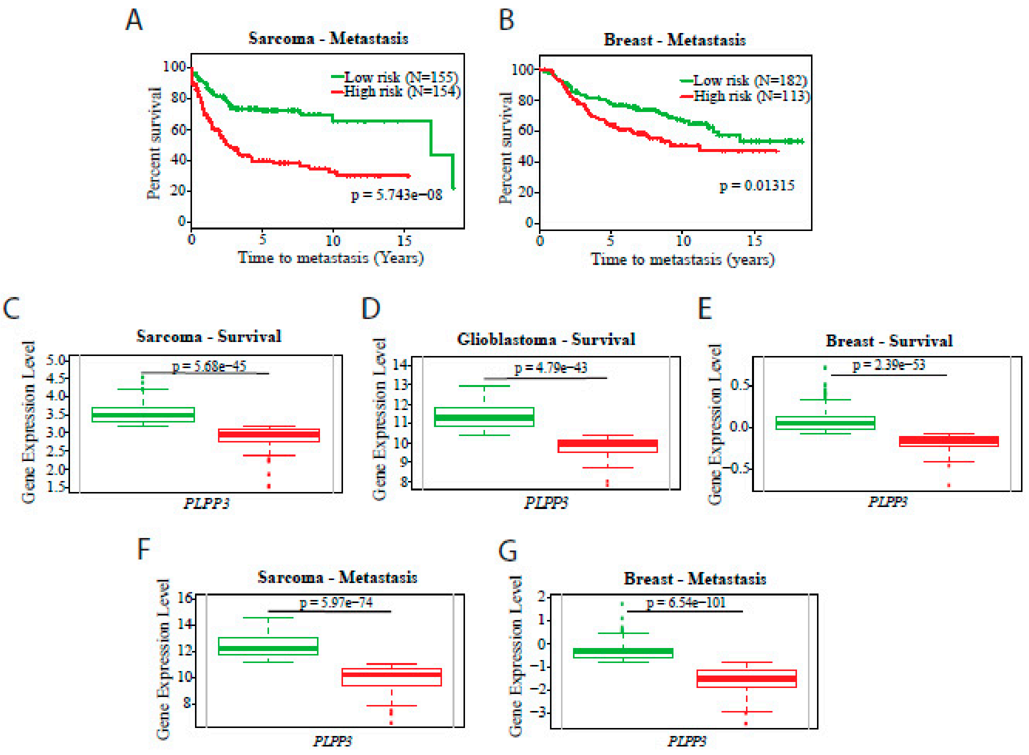

Supplementary Figure 2. Correlation between LPP3 gene expression and prognostic factors. (A-B) Kaplan-Meier plots obtained through SurvExpress online software showing metastasis-free survival curves of high- and low-prognostic risk groups based on *PLPP3* expression in **(A)** sarcoma and **(B)** breast cancer patient cohorts. Log-rank test p-values are presented. **(C-G)** *PLPP3* expression level associated with the high- (red) and low- (green) risk groups presented in Figure 2 **(M-O)** and Figure S2 **(A-B)**. p values were obtained using Student's t-test.

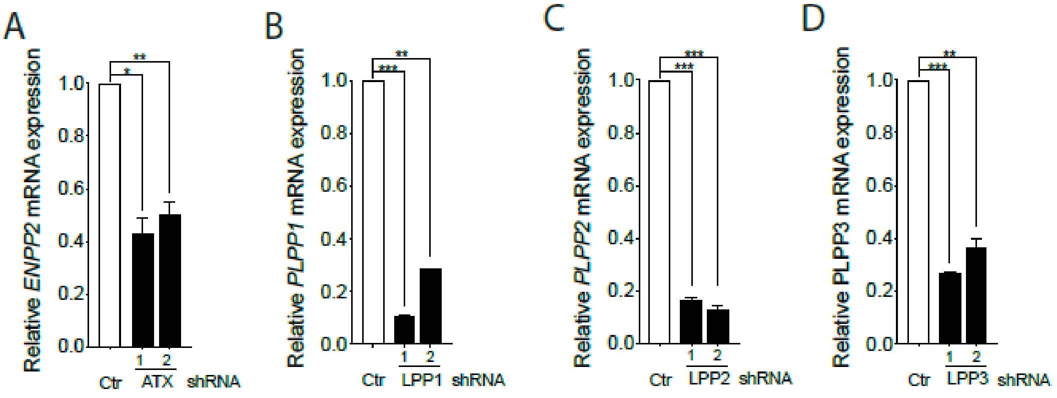

Supplementary Figure 3. Validation of ATX- and LPPs-targeted shRNAs by qPCR. (A-D) HT1080 cells were transfected with scramble, ATX-, LPP1-, LPP2-, or LPP3-targeted shRNAs. mRNA levels of **(A)** *ENPP2*, **(B)** *PLPP1*, **(C)** *PLPP2*, or **(D)** *PLPP3* were measured and quantified by qPCR. *RPLP0* was used to normalize the data (n=3). *Bars represent the mean ± SEM (* $P< 0.05$, ** $P< 0.01$, *** $P< 0.001$).*

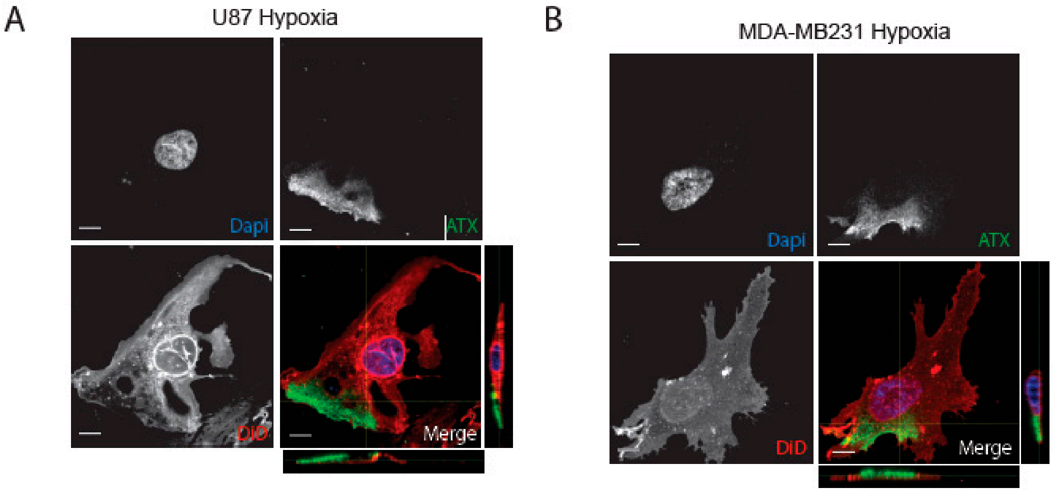

Supplementary Figure 4. ATX localization in hypoxic U87 and MDA-MB231 cell lines. (A-B) Representative immunofluorescence images of **(A)** U87 or **(B)** MD-MB231 cells cultured on non-fluorescent gelatin for 4h in hypoxia (1%O_2) are shown. Images show ATX (green) and DiD (red) staining in non-permeabilized cells. Nuclei were stained with DAPI (blue). Magnification 60X, scale bars, 5 μ m.

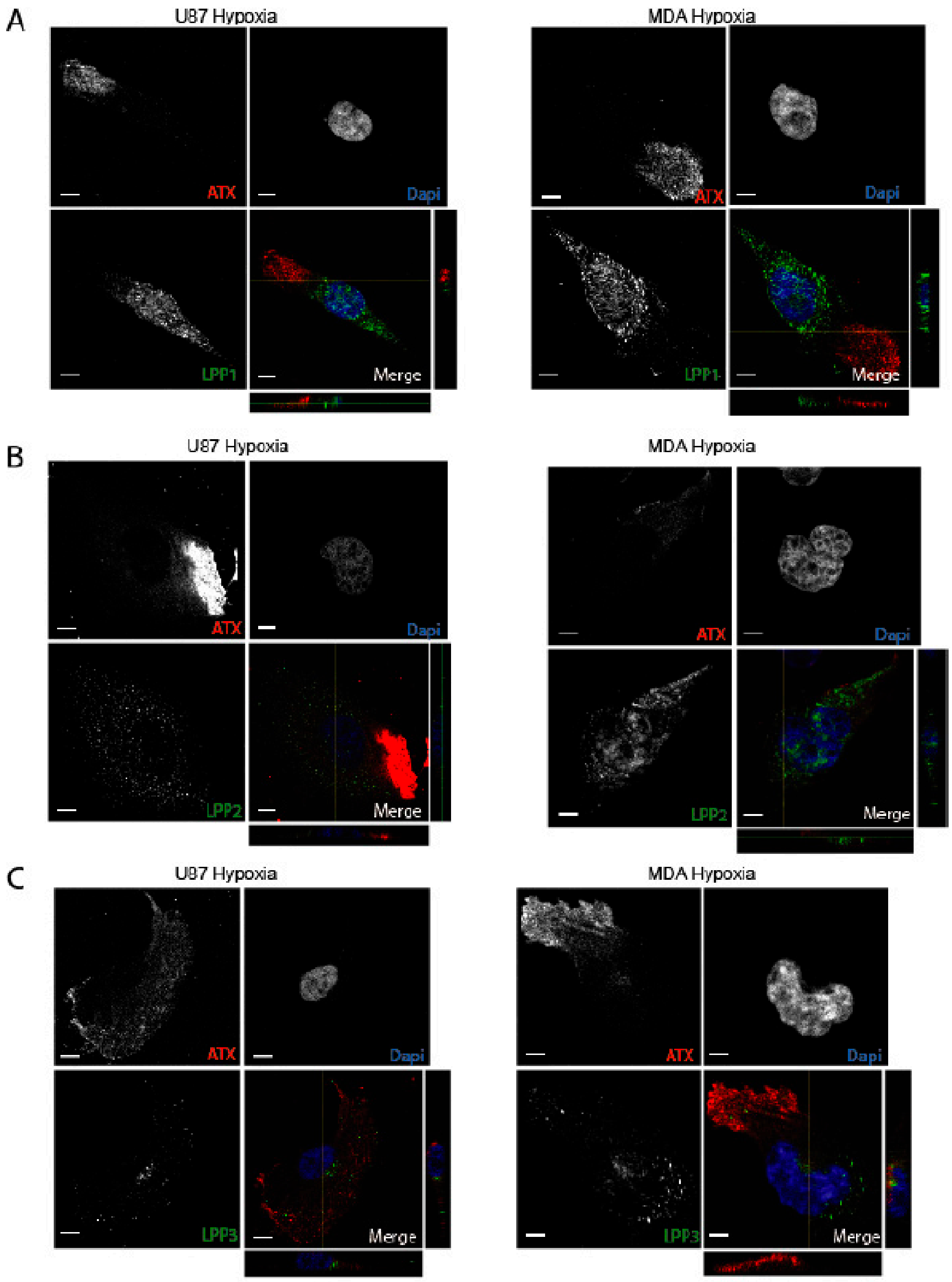

Supplementary Figure 5. Localization of both ATX and LPPs in hypoxic U87 and MDA-MB231 cell lines. (A-C) Representative immunofluorescence images of U87 or MDA-MB231 cells cultured on non-fluorescent gelatin for 4h in hypoxia (1%O_2) are shown. Cells were stained for **(A)** LPP1, **(B)** LPP2, or **(C)** LPP3 (green) and ATX (red). Nuclei were stained with DAPI (blue). Magnification 60X, scale bars, 5 μm.
